# Retinal blood flow association with age and weight in infants at risk for retinopathy of prematurity

**DOI:** 10.1038/s41598-024-63534-6

**Published:** 2024-06-04

**Authors:** Euna Cho, Urjita Das, Danielle Sidelnikov, Tara Balasubramanian, Daniel Shats, Shaiza Mansoor, He Eun Forbes, Jason Zhou, Ria Kapoor, Sera Chase, Madi Kore, Kristin Williams, Osamah Saeedi, Sripriya Sundararajan, Moran Roni Levin, Laurence Magder, Janet Leath Alexander

**Affiliations:** 1 University of Maryland School of Medicine, Baltimore, MD USA; 2 Department of Ophthalmology and Visual Sciences, University of Maryland School of Medicine, Baltimore, MD USA; 3 Division of Neonatology, Department of Pediatrics, University of Maryland School of Medicine, Baltimore, MD USA; 4 Department of Biostatistics and Epidemiology, University of Maryland, Baltimore, MD USA

**Keywords:** Medical research, Paediatric research

## Abstract

This prospective study evaluated the relationship between laser speckle contrast imaging (LSCI) ocular blood flow velocity (BFV) and five birth parameters: gestational age (GA), postmenstrual age (PMA) and chronological age (CA) at the time of measurement, birth weight (BW), and current weight (CW) in preterm neonates at risk for retinopathy of prematurity (ROP). 38 Neonates with BW < 2 kg, GA < 32 weeks, and PMA between 27 and 47 weeks underwent 91 LSCI sessions. Correlation tests and regression analysis were performed to quantify relationships between birth parameters and ocular BFV. Mean ocular BFV index in this cohort was 8.8 +/− 4.0 IU. BFV positively correlated with PMA (r = 0.3, *p* = 0.01), CA (r = 0.3, *p* = 0.005), and CW (r = 0.3, *p* = 0.02). BFV did not correlate with GA nor BW (r = − 0.2 and r = − 0.05, *p* > 0.05). Regression analysis with mixed models demonstrated that BFV increased by 1.2 for every kilogram of CW, by 0.34 for every week of CA, and by 0.36 for every week of PMA (*p* = 0.03, 0.004, 0.007, respectively). Our findings indicate that increased age and weight are associated with increased ocular BFV measured using LSCI in premature infants. Future studies investigating the associations between ocular BFV and ROP clinical severity must control for age and/or weight of the infant.

## Introduction

Retinopathy of prematurity (ROP) is the leading cause of preventable blindness in preterm infants in the US and worldwide^1,2^. The two most well-established risk factors for ROP are early gestational age (GA) and low birth weight (BW)^3–7^. The window of greatest risk for ROP progression dictates the timing of retinal screening eye examinations, which generally occur from 31 to 40 weeks or more of postmenstrual age (PMA).

The underlying pathogenesis of ROP is qualitatively described as an early ischemic phase followed by a late vasoproliferative phase^8,9^. Imaging tools that can capture high resolution and dynamic retinal blood flow patterns in premature infants offer promise in further elucidating the quantitative blood flow changes that occur during the phases of ROP. Tools currently available include laser speckle contrast imaging, color doppler imaging (CDI), intravenous fluorescein angiography, and optical coherence tomography (OCT) angiography.

Laser speckle contrast imaging (LSCI) permits the visualization and analysis of retinal blood flow dynamics in a noninvasive and non-contact manner^10–14^. Reflected laser illumination creates a “speckle” pattern during photography due to selective blurring from erythrocyte motion^11–13^. The selective blurring can be interpreted to simultaneously understand both the location and the speed of erythrocyte motion. Software creates a visual heatmap to visualize blood flow velocities within retinal vessels and numeric profiles of blood flow rates in a region of interest that can span one or more cardiac cycles. LSCI is particularly adaptable to infant imaging because it does not require the use of intravenous contrast agents, physical manipulation of the globe with a scleral depressor, a speculum, nor bright visible light, all of which are associated with stress, particularly in fragile preterm infants^15^.

Several previous studies have investigated ocular blood flow velocity (BFV) in neonates at risk for ROP^16,17^. Studies using CDI demonstrated a higher BFV in the ophthalmic artery and retinal vessels of infants with ROP compared to those without ROP^18–21^. Studies using OCT angiography have identified quantitative differences in tortuosity and vessel density associated with ROP^22,23^. Previous literature on LSCI has established an association between PMA and BFV^17^ and identified a decline in BFV following ROP treatment^24,25^, but no previous study had adequate power to formally test the hypothesis that birth parameters, age, and weight are associated with BFV. This study seeks to examine the association between LSCI-based BFV and five birth parameters: GA, PMA, chronological age (CA), BW, and current weight (CW) in preterm neonates at risk for ROP.

## Methods

The study was conducted in compliance with protocols approved by the accredited Institutional Review Board of the University of Maryland, Baltimore in adherence with the guidelines established by the US Health Insurance Portability and Accountability Act (HIPAA). Informed consent was obtained from the parent of each subject after explanation of the nature and possible consequences of the study.

### Participants

In this single-center prospective study, 38 neonates admitted to the Neonatal Intensive Care Unit (NICU) of the University of Maryland Children’s Hospital were included from May 2021 to October 2023. Eligible subjects met the following inclusion criteria: (1) the attending neonatologist requested ophthalmology consultation for ROP based on GA ≤ 30 weeks, or (2) BW ≤ 1500 g, (3) or BW 1500–2000 g or GA > 30 weeks with risk for ROP due to medically unstable clinical course^26^. Neonates were excluded from the study if they had media opacity, concurrent vascular or ocular disease other than ROP, a concurrent genetic syndrome, previous intraocular surgery, laser, or intravitreal pharmacologic treatment, or were otherwise evaluated as medically unstable per the attending neonatologist or bedside nurse. If eligible and amenable, the parent was invited to enroll their infant, and informed consent was obtained.

### Prior to imaging

Participants’ pupils were dilated with cyclopentolate hydrochloride 0.2%, phenylephrine hydrochloride 1% one hour before imaging. LSCI was scheduled concurrently with the indicated ROP screening eye examination so that pharmacologic dilation was not solely for the purposes of the research study. One drop of 0.5% proparacaine was placed in each eye immediately before the examination. A pulse oximeter linked to the LSCI device was placed on the participant’s foot. The infant was positioned supine, swaddled, and a 10-degree wedge was placed under the head and shoulders in all infants for comfort during exam. The wedge was selected because this allowed for optimal chin position during imaging and made infants with reflux more comfortable. The examining physician was at the infant’s head and the ROP nurse was at the infant’s feet. The eyelid of the eye was then gently opened by the gloved fingertips of the examining physician or ROP nurse. The nurse then tilted the head gently to the left or right to center the eye with the trajectory of the laser.

### Imaging

LSCI imaging was performed on a weekly basis, concurrent with standard ROP screening eye examination. Both eyes were imaged during each LSCI session, with the goal of one high quality image per eye per session. LSCI sessions were deferred if a subject was medically unstable. Retinal blood flow imaging with LSCI was conducted using the investigational XyCAM NEO prototype system (Vasoptic Medical, Inc., Columbia, MD, USA). The XyCAM prototype is an adaptation of the FDA-cleared XyCAM RI System (Vasoptic Medical, Inc., Columbia, MD, USA) that is mounted with a movable arm. The XyCAM NEO also has software customized for imaging retinal blood flow in supine infants in the NICU. XyCAM uses LSCI to obtain retinal blood flow data within a 25-degree field of view. In this study, the field of view was approximately centered on the optic nerve head by adjusting the relative positioning of the XyCAM with respect to the neonate’s eye. High frame-rate image data was acquired for duration of six seconds from each eye under near-infrared laser illumination (peak wavelength of 785 nm), with BFV index computation at every pixel. Focusing under laser illumination was assisted via visualization of blood flow velocities computed in real-time and depicted in pseudo-color^13^. The data was accepted if it contained a contiguous stretch of at least 2 s of 3 or greater artifact-free blood flow pulses (cardiac cycles) without significant motion of the eye. If spontaneous motion occurred, the image capture attempt was repeated up to 2 times, for a total XyCAM exam duration of no more than 10 min. Immediately following LSCI data collection protocol, the standard ROP eye screening exam was conducted with binocular indirect ophthalmoscopy followed by retinal photography using the RetCam 3 Shuttle (Natus Medical Inc., Middleton, WI, USA) if clinically indicated for advanced or progressive ROP.

### Image analysis

**Figure 1:**
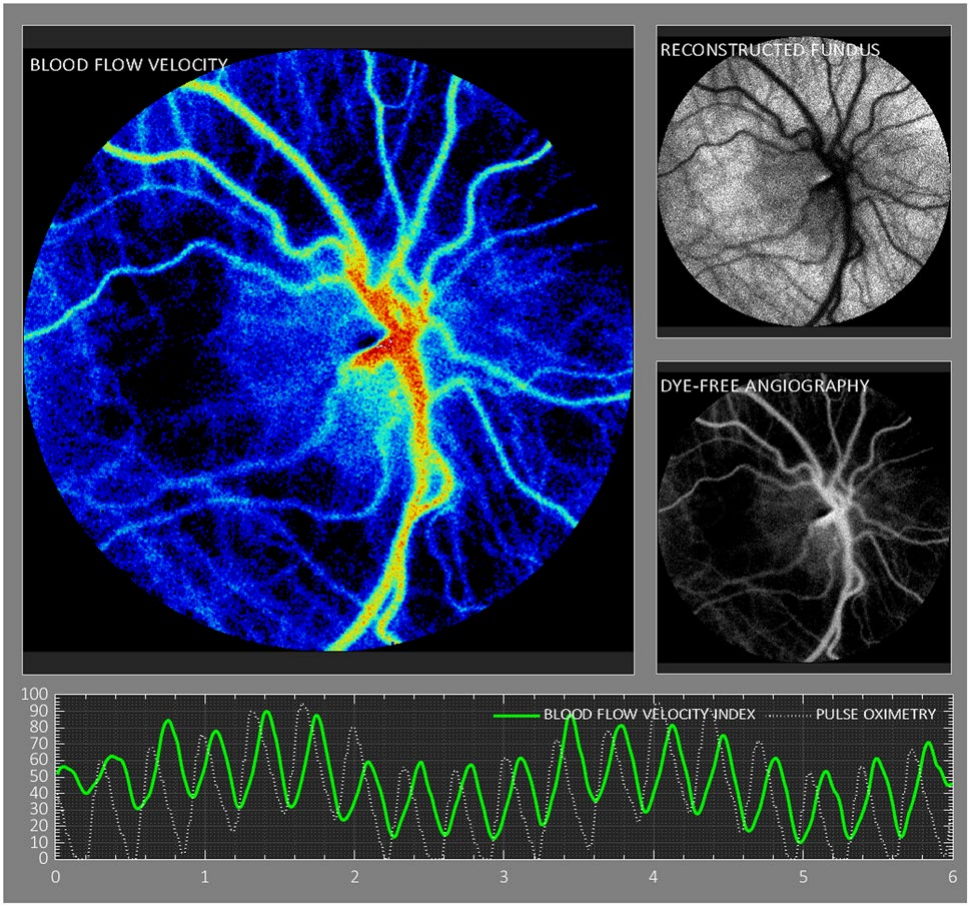
Image of ocular blood flow using laser speckle contrast imaging, demonstrating blood flow heatmap, reconstructed fundus image, and dye-free angiography. Bottom panel demonstrates blood flow velocity waveform with concurrent pulse oximetry tracing.

The XyCAM image analysis software provides the user with analytical tools to choose specific regions of interest, and select time-intervals over which the blood flow waveform will be analyzed. Detailed reports contain various retinal blood flow metrics associated with the user-chosen regions and time windows. To examine the retinal blood flow metrics at the optic nerve head, as a theoretical best indicator of total ocular blood flow, the outline of the optic nerve was drawn using a free-form region-selection tool while assisted by fundoscopic image overlay (Fig. 1). To compensate for any motion artifact, the analysis software registers all frames to a single image frame prior to computation of BFV indices^27^. The software also automatically detects peaks and dips in the blood flow waveform that can be manually adjusted if needed to define cardiac cycles. Time intervals chosen for analysis comprised of at least 2 continuous cardiac cycles, and the following 3 blood flow metrics were extracted and statistically analyzed: peak, mean, and dip BFV index. Final analysis was limited to the mean BFV index because results did not differ among peak, mean, and dip velocity indices.

### Data and statistical analysis

Blood flow indices were provided by the XyCAM NEO software and tracked by subject identification number and eye across serial examinations ranging from 1 to 6 imaging sessions per eye. Birth parameters (BW and GA) were collected from the history and physical in the electronic medical record on the day of birth. PMA and CA were calculated based on date of birth and GA. Current weight (CW) was collected on the same day as the eye exam or one-day prior nursing report in the electronic medical record. Univariate analysis was used to determine the means and distributions of data. Kendall’s correlation coefficients between birth parameters (GA, PMA, CA, BW, and CW) and XyCAM-derived waveform blood flow metrics were calculated based on a randomly selected single eye per subject (n = 38). Mixed-effects regression was used to model the association between blood flow and age and weight, while accounting for two eyes per subject and multiple imaging sessions per eye. The use of mixed models was applied to n = 91 exams and adjusted for repeat measures over time per eye. R Studio version 4.1.2 (R Foundation for Statistical Computing, Vienna) and SAS v9.4 (Statistical Analysis Software, North Caroline State University) were used to conduct the statistical analysis.

## Results

This study included 38 preterm infants who underwent 91 LSCI examinations. Table 1 provides demographic and clinical features of the cohort.

**Table 1:** Demographics and birth parameters.

Sex	53% Female (n = 20)		
Hispanic ethnicity	16% (n = 6)		
Race	34% Black (n = 13)		
	42% White (n = 16)		
	3% Asian (n = 1)		
	21% Other (n = 8)		

		Mean +/− SD	Range
Gestational age	n = 38 subjects	27.2 +/− 2.6 weeks	23.0–31.6
Birthweight		995 +/− 340 g	505–1915
Current age at imaging	n = 91 exams*	9.1 +/− 5.0 weeks	1.0–20.9
Current weight		2359 +/− 831 g	1100–4750

*56 Total exams per subject/eye; Each eye contributed 1.3 +/− 0.7 [range 1–4] different examination dates.

The cohort was 53% female, and 16% Hispanic ethnicity. Subject race was 34% Black, 42% White, 3% Asian, and 21% Other as identified by the mother. Mean gestational age was 27 +/− 3 and mean birthweight was 995 +/− 340 g. The mean age at imaging was 9.1 +/− 5 weeks and current weight at imaging was 2359 +/− 831 g.

**Figure 2:**
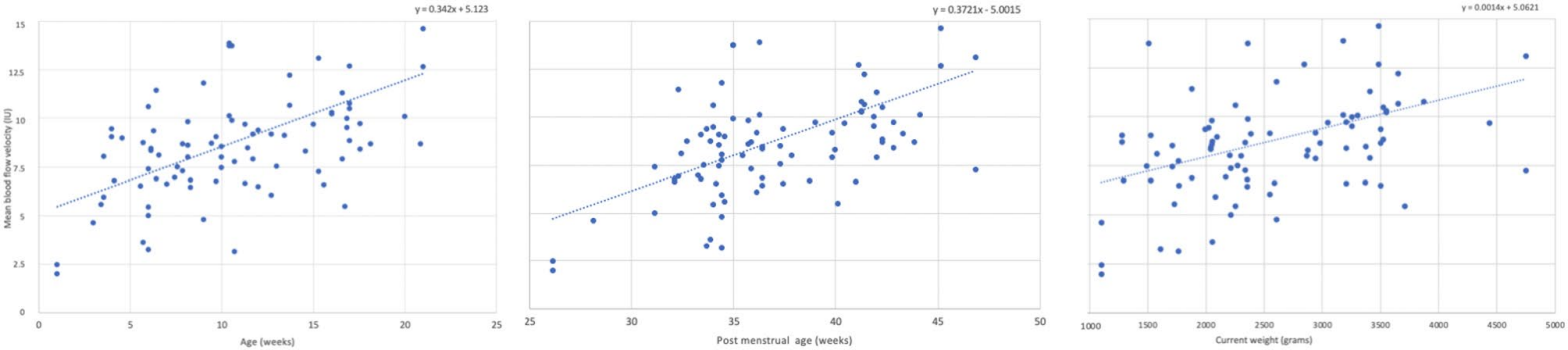
Scatterplot relationships for age, post menstrual age, and current weight with mean blood flow velocity.

The correlation between mean BFV and birth parameters are provided in Supplementary Table. The peak and dip blood flow velocities were the same as the mean BFV in correlation results, so the analysis only included mean BFV to avoid redundancy. Scatterplots demonstrating the association between mean BFV and birth parameters are provided in Fig. 2.

Mean BFV index in this cohort was 8.8 +/− 4.0 IU. The blood flow velocities were significantly associated with PMA, CA, and CW. BFV positively correlated with PMA (r = 0.3, *p* = 0.01), CA (r = 0.3, *p* = 0.005), and CW (r = 0.3, *p* = 0.02). BFV did not correlate with GA nor BW (r = − 0.2 and r = − 0.05, *p* > 0.05). CA was significantly correlated with GA and PMA. BW was significantly correlated with GA and PMA. CW was significantly correlated with PMA and CA. No significant correlation was observed for potential confounders sex (r = − 0.2, *p* = 0.3), race (r = − 0.02, *p* = 0.9), and heartrate (r = − 0.03, *p* = 0.8) with BFV.

Table 2 demonstrates the linear regression relationship between individual birth parameters and mean BFV while accounting for repeat measures from the same eye and the association between paired eyes (clustered data) of the same subject.

**Table 2:** Models for the association between age and weight with mean blood flow.

Mean BFV = [intercept] + 1.2084 Current weight (in kg)	***p* = 0.0280**
Mean BFV = [intercept] + 0.3382 Current age (in weeks)	***p* = 0.0004**
Mean BFV = [intercept] + 0.3640 Post menstrual age (in weeks)	***p* = 0.0007**
Mean BFV = [intercept] − 0.3522 Birthweight (in kg)	*p* = 0.8160
Mean BFV = [intercept] − 0.1766 Gestational age (in weeks)	*p* = 0.3841

Analysis used mixed models to simultaneously account for the inclusion of both eyes per subject, and the inclusion of repeat measures of the same eye (n = 91 eye exams). Bold indicates *p* < 0.05.

The regression analysis with mixed models demonstrated that mean BFV increased by 1.2 for every kilogram of weight gain, by 0.34 for every week of age, and by 0.36 for every week of PMA (*p* = 0.03, 0.0004, 0.0007, respectively). Mean BFV was not significantly associated with BW nor GA (*p* = 0.8 and 0.4, respectively). Each birth parameter variable was modeled separately due to collinearity among birth parameters (i.e. CA and PMA)*.*

## Discussion

This is the largest study to date to examine ocular blood flow associations with the age and weight of infants in an ROP screening cohort in the NICU setting. Our cohort included 38 infants from an American population of diverse backgrounds who underwent 91 separate eye imaging sessions. We confirmed the previously established association between PMA and ocular blood flow^17^. We further refined the understanding of age association with blood flow by identifying and quantifying the analogous association between chronological age and ocular blood flow. Similarly, we quantify the significant association between weight and ocular blood flow. Given that weight increases with age, these variables are colinear and therefore were evaluated in our model individually. Future studies may justify inclusion of one or more age or weight variables in models associating blood flow and ROP severity, depending on the sample size and objectives of the study.

The correlation between blood flow volume and the size or age of an individual is intuitive, but nuances in a population of premature infants may be influenced by the many unique features of their physiology including rapid growth rates (relative to adults), anemia of prematurity, exogenous fluids, and oxygen supplementation. While it would be impossible to adequately control for all these features, our cohort reflects a pragmatic population among whom all of these factors are present and likely relatively consistent.

Our results demonstrate that BFV changes in a predictable way during the early months of neonatal growth. Mean BFV index increases by 1.2 for every kilogram of weight gain, by 0.34 for every week of chronological age, and by 0.36 for every additional week of post menstrual age. Given that current age and post menstrual age are essentially the same variable, offset by gestational age, they cannot be meaningfully used in the same model. Similarly, age and weight are closely related and need not be included in the same model. In general, our results suggest that associations between blood flow and ROP severity will require controlling for at least one variable related to age or weight (postmenstrual age, current age, or current weight), and potentially all 3. Conversely, birthweight and gestational age did not show any meaningful association with mean BFV, and therefore should not be included in models evaluating ocular blood flow in ROP.

Previous literature on blood flow in ROP includes data from LSCI, CDI, and OCT-angiography^16,28–32^. Our results are consistent with prior work by Matsumoto et al., who reported significant association between post menstrual age and BFV as measured by LSCI^17^. Their group found a near significant association for current weight. They found no correlation of blood flow with chronological age but only with post menstrual age, possibly due to the choice of Pearson correlation rather than Kendall’s correlation, which can be unduly influenced by extreme values. Given the lack of normality and relatively small sample size, Kendall’s correlation was appropriate for our dataset. CDI studies are inconclusive regarding the relationship of blood flow with age or weight in an ROP screening cohort. Several studies using CDI found relationships between blood flow and ROP severity, but most did not evaluate the association between blood flow and age or weight^16,18,19,33^. Some CDI studies however did suggest increased blood flow with increasing age and weight, among infants in first week of life^20,21^. OCT angiography has similarly established quantitative vascular changes related to tortuosity and vessel area density in pre-term infants with more severe ROP^22^. No previous study using OCT angiography has evaluated the association between current age or weight and OCT angiographic features in infants at risk for ROP, Technical challenges limit clinical application of OCT in neonates because the tool is extremely sensitive to motion artifact. For example, previous literature using OCT angiography had sample size less than 10 infants^22,34^.

Although BW and GA are quite important in terms of ROP risk, we observed an absence of association with blood flow. This may be explained by the previous literature that indicates some contribution to ROP risk is also related to post-natal growth^35^. In terms of GA, we found that the chronological age has a stronger association with blood flow. A potential explanation is that blood volume and size of retinal vessels depends more upon the overall age of the infant than how early they were born. Although GA and BW are utilized as risk factors to determine which infants qualify for ROP screening, they are not predictive of retinal blood flow.

The strengths of our study include a diverse patient population, a relatively large sample size, and statistical analysis accounting for repeat measures. Our cohort was racially diverse, and we included high-risk neonates with much younger gestational ages (as young as 23 weeks) and extremely low birthweights (505 g), compared to prior literature. Furthermore, our cohort was mostly incubator-bound and the majority of subjects were on respiratory support or supplemental oxygen. In this way, our study reflects a “real world” ROP cohort. This study also offers the largest sample size to date, compared to previous ROP studies using laser speckle contrast imaging. Our study included 38 subjects and 91 eye exams. We analyzed both eyes per subject and included repeat measures. We accounted for the inclusion of both eyes and repeat measures statistically. Our use of mixed models to account for repeat measures offers more power to detect relationships between variables that vary over time, highly appropriate to this case looking at age and weight.

The limitations of this study included a challenging population of awake neonates, with slightly inferior reproducibility compared to adults^17,36^. This study excluded neonates with significant systemic medical instability, but the cohort did include some infants with a history of various common comorbidities of prematurity (i.e. bronchopulmonary dysplasia), however we lacked adequate sample size to control for these comorbidities. Although our study examined the association of heart rate with BFV, we lacked reliable systemic blood pressure data. Previous related work did not find any significant association between blood pressure and blood flow in similar cohorts^17,18^, as justification for the lack of inclusion of blood pressure in the data collection and analysis. If a future analysis includes a comparison of treatment-warranted ROP, it will be necessary to evaluate the contribution of sex, race, and heart rate in the model. Lastly, while LSCI provides indices of blood flow that are repeatable and reproducible^11,13,14,27,36^, it has never been validated to measure absolute blood in vivo in human eyes. Therefore, though comparisons can be made across subjects measured on the same device and relative changes can be compared across devices, direct comparisons are not possible between different LSCI devices. This differs from CDI, where absolute flow velocity in mm/s is available but not at a comparable resolution as LSCI. LSCI has key advantages over CDI including improved resolution, non-contact imaging, and ability to evaluate more anatomic regions of the retina. Also, LSCI offers concurrent retinal imaging and retinal blood flow evaluation; while CDI is limited to retrobulbar blood flow assessment.

This study is a preliminary and prerequisite step in exploring the relationship between ROP and blood flow. Due to small sample size, we limited our analysis to variables we had power to evaluate, namely age and weight. Future studies may investigate a larger sample size to better assess associations between cardiopulmonary comorbidities, hemoglobin, oxygen saturation, and other risk factors that might affect BFV. In addition, it is suggested to investigate whether there is an association between hemoglobin level and retinal blood flow, and if there are differences with recent transfusions, which pertinent to premature infants.

Our findings indicate that increased age and weight are associated with increased ocular BFV measured using LSCI in premature infants. This work highlights the importance of controlling for the age and/or weight of the infant in future studies investigating associations between ocular BFV and ROP clinical severity.

### Supplementary Information

Supplementary Tables.

## Data Availability

The de-identified participant (including data dictionaries) dataset generated during and analyzed during the current study are available from the corresponding author on reasonable request.
