# Supplementary material for: Retinal blood flow association with age and weight in infants at risk for retinopathy of prematurity
Source: Sci Rep. 2024 Jun 4;14:12790. doi: 10.1038/s41598-024-63534-6 (PMC11150459; doi:10.1038/s41598-024-63534-6)
Supplement: Supplementary file 1 — Supplementary Tables. [file 41598_2024_63534_MOESM1_ESM.docx]

Supplemental Table. Kendall Tau-B correlation coefficients and hypothesis-values for n=38 participants with analysis of a single observation and single eye per subject. (GA=gestational age, PMA=post-menstrual age, CA=chronological age, BW=birthweight, CW=current weight, BFV= mean blood flow velocity index in arbitrary units). **Bold** indicates p<0.05.

|  | GA | PMA | CA | BW | CW | BFV |
| --- | --- | --- | --- | --- | --- | --- |
| GA |  | \| 0.08087 \| \| --- \| \| 0.4806 \| | \| -0.48012 \| \| --- \| \| **<.0001** \| | \| 0.60809 \| \| --- \| \| **<.0001** \| | \| -0.12528 \| \| --- \| \| 0.2733 \| | \| -0.14954 \| \| --- \| \| 0.1904 \| |
| PMA |  |  | \| 0.44971 \| \| --- \| \| **<.0001** \| | \| 0.02147 \| \| --- \| \| 0.8503 \| | \| 0.65522 \| \| --- \| \| **<.0001** \| | \| 0.27857 \| \| --- \| \| **0.0142** \| |
| CA |  |  |  | \| -0.36846 \| \| --- \| \| **0.0012** \| | \| 0.55015 \| \| --- \| \| **<.0001** \| | \| 0.31617 \| \| --- \| \| **0.0054** \| |
| BW |  |  |  |  | \| -0.00428 \| \| --- \| \| 0.9699 \| | \| -0.05132 \| \| --- \| \| 0.6508 \| |
| CW |  |  |  |  |  | \| 0.26353 \| \| --- \| \| **0.0200** \| |
